# Validation of the 5-domain Niemann-Pick type C Clinical Severity Scale

**DOI:** 10.1186/s13023-021-01719-2

**Published:** 2021-02-12

**Authors:** Marc C. Patterson, Lucy Lloyd-Price, Christina Guldberg, Helen Doll, Claire Burbridge, Michael Chladek, Christine íDali, Eugen Mengel, Tara Symonds

**Affiliations:** 1 Mayo Clinic Children’s Center, 200 1st St SW, Rochester, MN 55905 USA; 2 Clinical Outcomes Solutions, Folkestone, Kent UK; 3 Orphazyme A/S, Copenhagen, Denmark; 4 Clinical Outcomes Solutions, Chicago, IL USA; 5 SphinCS GmbH, Institute of Clinical Science for LSD, Hochheim, Germany

**Keywords:** Niemann-pick type C clinical severity scale, NPCCSS, ClinRO, Validation, Arimoclomol

## Abstract

**Background:**

Niemann-Pick disease type C (NPC) is an ultra-rare, progressive, genetic disease leading to impaired lysosomal function and neurodegeneration causing serious morbidity and shortened life expectancy.

The Niemann-Pick type C Clinical Severity Scale (NPCCSS) is a 17 domain, disease-specific, clinician-reported outcome measure of disease severity and progression. An abbreviated 5-domain NPCCSS scale has been developed (measuring Ambulation, Swallow, Cognition, Speech, and Fine Motor Skills) and the scale reliability has been established. Additional psychometric properties and meaningful change of the scale need, however, to be assessed.

**Methods:**

Mixed method studies were conducted to ascertain which NPCCSS domains were most important, as well as to explore meaningful change: 1) surveys in caregivers/patients (n = 49) and 2) interviews with clinicians (n = 5) as well as caregivers/patients (n = 28). Clinical trial data (n = 43) assessed construct validity and meaningful change through an anchor-based approach.

**Results:**

Domains identified as most important by clinicians, caregivers, and patients (independent of current age, age of onset, and disease severity) were Ambulation, Swallow, Cognition, Speech, and Fine Motor Skills, indicating content validity of the 5-domain NPCCSS.

Criterion validity was shown with the 5-domain NPCCSS being highly correlated with the 17-item NPCCSS total score (excluding hearing domains), r^2^ = 0.97. Convergent validity was demonstrated against the 9 Hole Peg Test, r^2^ = 0.65 (n = 31 patients), and the Scale for Assessment and Rating of Ataxia (SARA), r^2^ = 0.86 (n = 49 patients). Any change was seen as meaningful by patients/caregivers across domains. Meaningful change using trial data and interviews with NPC experts (n = 5) and patients/caregivers (n = 28) suggested that a 1-category change on a domain is equivalent to 1-point change or greater in the 5-domain NPCCSS total score.

**Conclusions:**

Qualitative and quantitative data support content and construct validity of the 5-domain NPCCSS score as a valid endpoint in NPC trials. A 1-category change on any domain is equivalent to 1-point change or greater in the 5 domain NPCCSS total score, representing a clinically meaningful transition and reflecting loss of complex function and increased disability.

*Trial registration* NCT02612129. Registered 23 November 2015, https://clinicaltrials.gov/ct2/show/NCT02612129

## Background

Niemann-Pick disease type C (NPC) is a rare, progressive, neurodegenerative disease arising from autosomal recessive mutations in the *NPC1* (≈95% of cases) or *NPC2* (≈5% of cases) genes [1, 2], which encode essential lysosomal proteins associated with intracellular lipid transport and metabolism [3, 4]. Mutated NPC proteins are often misfolded and degraded prematurely, leading to impaired lysosomal function, accumulation of multiple lipid species, and neurodegeneration accompanied by disease symptoms of the liver, spleen, and lungs [4, 5]. The age of onset for NPC disease can vary greatly, from a neonatal, rapidly fatal disorder to an adult-onset, slowly progressing neurodegenerative disease. NPC has an estimated incidence of approximately 1:100,000 live births [6]. There are limited treatment options for NPC and the only approved drug in Europe is miglustat.

The NPC Clinical Severity Scale (NPCCSS) is a disease-specific, clinician-reported outcome (ClinRO) measure that was developed to characterize and quantify disease progression. [7] The original NPCCSS has 9 major domains and 8 minor domains. Four of the major domains (Ambulation, Fine Motor Skills, Swallow, and Speech) were modified from a disability scale developed by Iturriaga et al., [8] (Seizures and Ocular movements were later added to this scale [9]); additional clinical findings frequently observed in NPC (Cognition, Eye Movement, Hearing, Memory, and Seizures) were added to form the 9 major domains. These reflect the major neurological features of NPC. Other important but less frequent clinical aspects of NPC were also included as minor domains (Auditory Brainstem Response, Behavior, Gelastic Cataplexy, Hyperreflexia, Incontinence, Narcolepsy, Psychiatric, and Respiratory Status).

The NPCCSS has shown good inter-rater reliability [7, 10], construct validity, and internal consistency [7]. Responsiveness of the NPCCSS has also been demonstrated in 2 published studies [11, 12].

The NPCCSS allows a comprehensive assessment of the symptom burden experienced by NPC patients. However, in clinical research, a short form version would be beneficial, allowing clinicians to focus on the core symptoms of the condition, evaluate outcome across those specific symptoms and reduce variability, as well as increase feasibility in studies. Additionally, a shortened version would be ideal for use in daily practice research as it is less time consuming for clinicians to complete. Therefore a 5-domain version has been developed to cover Cognition, Swallow, Fine Motor Skills, Speech, and Ambulation (Table 1). The domain choice was based on the original work by Iturriaga et al., [8] which included 4 of the 5 domains, and cognition was added due to its importance as identified by Cortina-Borja et al. [12] These core domains were also highlighted as the most important from a survey conducted at a recent Patient Focused Drug Development meeting in the US [13]. Criterion validity of the 5-domain version in terms of its correlation with the 17-domain NPCCSS has been shown [12] and reliability of the 5-domain measure has also been established, with both strong intra-rater reliability and inter-rater reliability being demonstrated [14].

**Table 1:** 5-domain NPCCSS

Domain	Scoring	Minimum–Maximum Score
Ambulation	0 = Normal 1 = Clumsy 2 = Ataxic unassisted gait or not walking by 18 months 4 = Assisted ambulation or not walking by 24 months 5 = Wheelchair dependent	0–5
Fine Motor Skills	0 = Normal 1 = Slight dysmetria/dystonia (independent manipulation) 2 = Mild dysmetria/Dystonia (requires little to no assistance, able to feed self without difficulty) 4 = Moderate dysmetria/dystonia (limited fine motor skills, difficulty feeding self) 5 = Severe dysmetria/Dystonia (gross motor limitation, requires assistance for selfcare activities)	0–5
Swallow	0 = Normal, no dysphagia 1 = Cough while eating Intermittent dysphagia* + 1 = w/Liquids + 1 = w/Solids Dysphagia* + 2 = w/Liquids + 2 = w/Solids 4 = Nasogastric tube or gastric tube for supplemental feeding 5 = Nasogastric tube or gastric tube feeding only	0–5
Cognition	0 = Normal 1 = Mild learning delay, grade appropriate for age 3 = Moderate learning delay, individualized curriculum or modified work setting 4 = Severe delay/plateau, no longer in school or no longer able to work, some loss of cognitive function^^^ 5 = Minimal cognitive function	0–5
Speech	0 = Normal 1 = Mild dysarthria (easily Understood 2 = Severe dysarthria (difficult to understand) 3 = Non-verbal/functional communication skills for needs 5 = Minimal communication	0–5
5-domain NPCCSS score	Sum of all scores from the 5 domains above	0–25 (higher score = more severe clinical impairment)

^*^Score is additive (to the “cough while eating”-score of 1) within the two subsections of intermittent dysphagia and dysphagia (example: for intermittent dysphagia with solids and dysphagia with liquids a score of 4 applies (1 + 1 + 2))

To further investigate the 5-domain version of the NPCCSS (Table 1), 2 studies were undertaken to evaluate the relevance of these 5 core domains from a patient, caregiver, and clinician perspective. These studies also sought to explore how patients or caregivers and clinicians would define meaningful change across these 5 domains. Data from a clinical trial [15] was used to assess the construct validity, sensitivity to change, and meaningful change threshold of the 5-domain NPCCSS using an anchor-based approach (Clinician Global Impression of Improvement [CGI-I]) as recommended by regulatory agencies.

## Results

### Study population

#### NPC survey (patients/caregivers)

In this study (OR-SRV-NPC-01), NPC patient advocacy groups in the US and UK advertised the study to their members, with 49 completed surveys (n = 37 US and n = 12 UK) included in the final analyses: n = 43 family caregivers (reporting for n = 22 paediatric patients and n = 21 adult patients) and n = 6 adult patients reporting for themselves. Twenty-eight of these survey respondents also took part in a follow-up telephone interview (n = 20 US and n = 8 UK, made up of n = 5 adult patients and n = 23 caregivers). Overall, a broad range of ages and levels of NPC disease severity were included. Patient participants represented a pediatric population and an adult population: range 13 months to 65 years; mean (SD) current age 8.1 (5.5) years (median 8.0 years) and 33.3 (14.2) years (median 30.0 years), respectively. The disease severity ranged from ≤ 4 (n = 7); 5–19 (n = 36); and ≥ 20 (n = 6) on the 5-domain NPCCSS. The patients in the survey and interviews were similar in age, age at symptom onset, and disease severity (Table 2).

**Table 2:** NPC survey sample characteristics by Country

Sample characteristic		Surveys			Interviews		
		US	UK	Total	US	UK	Total
Total	n (% of row total)	37 (75.5)	12 (24.5)	49	23 (71.9) (20 [71.4])^a^	9 (28.1) (8 [28.6])^a^	32^a^
Current age n (% of column total)	6 months to < 4 years^*^	5 (13.5)	0	5 (10.2)	1 (4.3)	0	1 (3.1)
	4–17 years	13 (35.1)	4 (33.3)	17 (34.7)	7 (30.4)	3 (33.3)	10 (31.3)
	≥ 18 years	19 (51.4)	8 (66.7)	27 (55.1)	15 (65.2)	6 (66.7)	21 (65.6)
Reported age of onset of first NPC related symptom n (% of column total)	< 3 months	6 (16.2)	2 (16.7)	8 (16.3)	5 (21.7)	1 (11.1)	6 (18.8)
	3 months to < 2 years	7 (18.9)	0	7 (14.3)	3 (13.0)	0	3 (9.4)
	2 to < 6 years	6 (16.2)	1 (8.3)	7 (14.3)	2 (8.7)	0	2 (6.3)
	6 to 15 years	10 (27.0)	4 (33.3)	14 (28.6)	8 (34.8)	4 (44.4)	12 (37.5)
	> 15 years	8 (21.6)	5 (41.7)	13 (26.5)	5 (21.7)	4 (44.4)	9 (28.1)
5-domain NPCCSS Score Total n (% of column total)	≤ 4	6 (16.2)	1 (8.3)	7 (14.3)	5 (21.7)	1 (11.1)	6 (18.8)
	5 to 19	29 (78.4)	7 (58.3)	36 (73.5)	17 (73.9)	6 (66.7)	23 (71.9)
	≥ 20	2 (5.4)	4 (33.3)	6 (12.2)	1 (4.3)	2 (22.2)	3 (9.4)

^a^ The interviews are counted by patient, however 4 caregivers (3 in US and 1 in UK) interviewed had 2 children in the family with NPC. Number of participants interviewed indicated in parentheses

^*^Youngest participant 13 months old

Adult participants experienced their first NPC symptom later in life as compared to the younger age groups (Table 3).

**Table 3:** Age at symptom onset by current age

Age at symptom onset (n) Current age (n)	< 3 months	3 months—< 2 years	2 years—< 6 years	6 years -15 years	> 15 years	Total
6 months to < 4 years	1	3	1	0	0	5
4–17 years	5	3	4	3	0	15
≥ 18 years	1	2	2	11	13	29
Total	7	8	7	14	13	49

#### NPC expert interviews

For the second study (OR-SRV-NPC-03), 5 NPC clinicians (n = 2 based in US and n = 3 in Europe) participated in an interview. All the clinicians met the eligibility criteria of 5 years plus experience with clinical evaluation and management/treatment of NPC patients and clinical expertise, thus were considered to be experts in NPC.

### Most important domains

#### NPC survey findings

When ranking the top 5 most important domains, Ambulation was most frequently chosen. Other domains frequently chosen (ie, by > 20 participants) were Swallow, Speech, Memory, Cognition, and Fine Motor Skills. Table 4 outlines the frequency with which each domain was ranked in the top 5 most important domains.

**Table 4:** Ranking of 5 most important domains in the web survey

	# Times ranked n (% of row total)					# Times in Top 5 n (% of total participants [n = 49])
	First	Second	Third	Fourth	Fifth	
Ambulation	10 (24.4)	12 (29.3)	9 (22.0)	4 (9.8)	6 (14.6)	41 (83.7)
Swallow	8 (22.9)	6 (17.1)	8 (22.9)	8 (22.9)	5 (14.3)	35 (71.4)
Speech	1 (3.1)	4 (12.5)	9 (28.1)	8 (25.0)	10 (31.3)	32 (65.3)
Memory	3 (10.7)	4 (14.3)	5 (17.9)	9 (32.1)	7 (25.0)	28 (57.1)
Cognition	8 (34.8)	4 (17.4)	4 (17.4)	4 (17.4)	3 (13.0)	23 (46.3)
Fine motor skills	2 (9.1)	8 (36.4)	2 (9.1)	5 (22.7)	5 (22.7)	22 (44.9)
Eye movement	2 (10.5)	3 (15.8)	5 (26.3)	2 (10.5)	7 (36.8)	19 (38.8)
Seizures	8 (57.1)	1 (7.1)	0	3 (21.4)	2 (14.3)	14 (28.6)
Hearing	2 (18.2)	3 (27.3)	3 (27.3)	1 (9.1)	2 (18.2)	11 (22.4)

The five domains of the 5-domain NPCCSS were included in the 6 most frequently selected domains. When comparing across current age groups (Table 5), these were consistently ranked across all groups at a similar rate except for Fine Motor Skills, which was less frequently included in the 5 most important symptoms by caregivers of patients 4–17 years old (23.5%) than by adult patients or their caregivers (59.3%). A similar discrepancy with Fine Motor Skills was found when comparing across groups according to age at first symptom onset, with this being less frequently reported as 1 of the 5 most important symptoms for those aged 3–23 months (first symptom onset) (28.6%) and 6–15 years (28.6%), however those in the less than 3 months (50%) and 2–5 years groups (57.1%) were similar to those aged older than 15 years (first symptom onset) (61.5%) (Table 6).

**Table 5:** Frequency of ranking domain in top 5 by current patient age

n (%)	6 months–3 years (n = 5)	4–17 years (n = 17)	≥ 18 years (n = 27)	Overall (n = 49)
Ambulation	4 (80.0)	13 (76.5)	24 (88.9)	41 (83.7)
Fine motor skills	2 (40.0)	4 (23.5)	16 (59.3)	22 (44.9)
Speech	3 (60.0)	11 (64.7)	18 (66.7)	32 (65.3)
Swallow	4 (80.0)	11 (64.7)	20 (74.1)	35 (71.4)
Cognition	2 (40.0)	9 (52.9)	12 (44.4)	23 (46.9)

**Table 6:** Frequency of ranking domain in top 5 by age of patient's first symptom

n (%)	< 3 months (n = 8)	3–23 months (n = 7)	2–5 years (n = 7)	6–15 years (n = 14)	> 15 years (n = 13)	Overall (n = 49)
Ambulation	8 (100.0)	4 (57.1)	6 (85.7)	12 (85.7)	11 (84.6)	41 (83.7)
Fine motor skills	4 (50.0)	2 (28.6)	4 (57.1)	4 (28.6)	8 (61.5)	22 (44.9)
Speech	3 (37.5)	4 (57.1)	7 (100.0)	8 (57.1)	10 (76.9)	32 (65.3)
Swallow	5 (62.5)	6 (85.7)	6 (85.7)	11 (78.6)	7 (53.8)	35 (71.4)
Cognition	6 (75.0)	4 (57.1)	2 (28.6)	6 (42.9)	5 (38.5)	23 (46.9)

When comparing across NPC severity groups, Fine Motor Skills and Swallow were less frequently included in the top 5 of importance in the low severity groups; conversely, the high severity group all included Swallow in their top 5 (Table 7)

**Table 7:** Frequency of ranking symptom in top 5 by disease severity of patient

n (%)	Disease severity based on responses to patient/caregiver reported 5-domain NPCCSS in the survey			Overall (n = 49)
	≤ 4 (n = 7)	5–19 (n = 36)	≥ 20 (n = 6)	
Ambulation	6 (85.7)	31 (86.1)	4 (66.7)	41 (83.7)
Fine motor skills	2 (28.6)	17 (47.2)	3 (50.0)	22 (44.9)
Speech	4 (57.1)	26 (72.2)	2 (33.3)	32 (65.3)
Swallow	3 (42.9)	26 (72.2)	6 (100.0)	35 (71.4)
Cognition	5 (71.4)	16 (44.4)	2 (33.3)	23 (46.9)

### Interview findings

#### Patient/caregiver

The reasons most often cited for a particular domain being identified in the top 5 were: the importance to patients of being able to move independently or not relying on someone to be mobile, and concerns about risks of falling or the greater health impacts associated with losing the ability to walk (Ambulation); the impact hand tremors or difficulty coordinating their hands had on their everyday activities like eating, writing, and caring for themselves (Fine Motor Skills); the importance communication has in everyday life, especially in expressing their needs or wants (Speech); the possible risks of choking, aspiration, pneumonia, and even death (Swallow); and the impact on patients’ education or work (Cognition and Memory). Although they are captured distinctly in the measure, some participants expressed in interviews that they saw cognition and memory as highly inter-related and, therefore, said that it was difficult to rank one as more important than the other.

#### NPC expert interviews

All 5 NPC expert clinicians were familiar with the original 17 domain NPCCSS scale and agreed that the 5-domain NPCCSS captures the core symptoms of NPC, and probably those that are most important to patients and their families as they affect the patients’ quality of life and functioning the most. Two clinicians highlighted that the symptoms captured in the 5-domain version are present in most patients with NPC, whereas the other NPC symptoms captured in the full 17 domain measure are not.

### Meaningful change threshold findings

#### Interview findings: patient/caregiver

Given the progressive nature of NPC, any level of change in any symptom was often seen by caregivers and patients as meaningful because it was an indication to them that their NPC was further progressing and worsening. In relation to what degree of change (specifically worsening) from their current level of severity would be meaningful to them in each domain of the 5-domain NPCCSS, the vast majority (61.9%–88.5%) of participants indicated that a 1-category worsening in each of the domains would be a meaningful deterioration. The feedback per domain is shown in Table 8.

**Table 8:** Level of worsening considered meaningful across 5-domain NPCCSS

	1-category meaningful, n (%)		2-categories meaningful (if 1-category not meaningful), n (%)	
	Yes	No	Yes	No
Ambulation (n = 26)	23 (88.5)	3 (11.5)	3 (100)	0
Fine Motor Skills (n = 25)	22 (88.0)	3 (12.0)	3 (100)	0
Speech (n = 26)	21 (80.8)	5 (19.2)	4 (100)	0
Swallow (n = 23)	17 (73.9)	6 (26.1)	5 (83.3)	1 (16.7)
Cognition (n = 21)	13 (61.9)	8 (38.1)	5 (100)	0

Additionally, nearly all participants expressed during interviews that slowing the progression of any one of the domains in the 5-domain NPCCSS would be meaningful. The 2 most important domains to slow the progression were considered to be Ambulation and Cognition.

#### NPC expert interviews

There were mixed responses to the question of what specific change in score on the 5-domain NPCCSS is considered meaningful, as clinicians felt that this depended upon patient age, NPC severity, and the domain changing. However, most of the clinicians considered a 1-category change to be meaningful, corresponding to a 1- or 2-point change within a domain of the 5-domain NPCCSS. There were individual differences among clinicians in which domains would be most meaningful to change; however, overall, the clinicians felt that change in any of the domains and delaying progression would be important.

#### NPC clinical trial

In a placebo-controlled clinical trial (CT-ORZY-NPC-002) based on the CGI-I (between baseline and end of treatment), patients who were reported to have “no change” in their condition (n = 18) had a mean (SD) change in the 5-domain NPCCSS total score of 0.83 (2.176) with a median change of 0. The upper 95% CI of those who reported no change was 1.915. The collapsed “worsening” category on the CGI (n = 13) had a mean (SD) change in the 5-domain NPCCSS total score of 2.69 (3.225) with a median change of 2 points (Table 9).

**Table 9:** Summary table of anchor-based NPCCSS results for the CGI-I

Visit	Collapsed / Uncollapsed	Anchor Category	N	Mean Change (SD)	95% CI	Effect Size	Median Change
Visit 1 to 6	Collapsed	No Change	18	0.83 (2.176)	(-0.249, 1.915)	0.38	0.00
		Worsening	13	2.69 (3.225)	(0.744, 4.641)	0.83	2.00

Abbreviations: CGI-I = Clinician Global Impression of Improvement; CI = confidence interval; NPCCSS = Niemann-Pick type C Clinical Severity Scale; SD = standard deviation

Visit 1 = Screening; Visit 3 = 3 months; Visit 4 = 6 months; Visit 5 = 9 months; Visit 6 = 12 months

All patients in the study (age range 2–19 years) were assessed using the CGI-I

#### Construct validity

Convergent validity was demonstrated at baseline both between Fine motor skills versus the 9 Hole Peg Test [16] and between the 5-domain NPCCSS total score versus the 9 Hole Peg Test, [16] both r^2^ = 0.65 (n = 31 patients), and between the 5-domain NPCCSS total score versus Scale for Assessment and Rating of Ataxia (SARA), [17] r^2^= 0.86 (n = 49 patients).

#### Criterion validity

The 5-domain NPCCSS also correlated highly with the total score of the 17-item NPCCSS (excluding hearing domain), r^2^  = 0.97 (n = 49 patients), demonstrating a high level of criterion validity.

#### Sensitivity to change

In the clinical trial [15] the 15 patients with non-missing data in the placebo group deteriorated in score at 12 months by a mean of 2.14 points (range − 1 to + 11) while the 27 patients with non-missing data in the arimoclomol group deteriorated by a mean of 0.7 (range -2 to + 7), giving an overall treatment effect of − 1.34 (95% CI − 2.71 to 0.02; p = 0.0537).

## Discussion

The original 17 domain NPCCSS was developed to characterize and quantify disease progression across all symptoms experienced by NPC patients. However, a smaller number of items in a measure can reduce variability and provide a more focused subset of symptoms. Focusing on the most relevant domains as assessed by patients, caregivers, and clinicians (Ambulation, Fine Motor Skills, Swallow, Cognition, and Speech), will help inform treatment effects where there are such effects. An abbreviated tool is also helpful in daily clinical practice. From the mixed methods approach used in the current research, involving both patients and caregivers, and the qualitative clinician interviews, the 5-domain NPCCSS was found to be content valid and suitable for use across ages, age of onset, and severity subgroups. This is supported by prior research from Iturriaga et al. [8] and Cortina-Borja et al., [12] and is consistent with the report from a recent Patient Focused Drug Development workshop. [13].

Difficulties with ambulation, swallowing, and speech were highly salient NPC symptoms to the participants interviewed. After these 3 symptoms, Fine Motor Skills, Cognition, and Memory were those most often discussed by participants as being important because of the impacts on patients and caregivers. Cognition and Memory were seen by many participants as being highly interrelated if not the same thing, thus use of one or other in a grouping of the most important symptoms appears to be acceptable.

The 5-domain NPCCSS was shown to have construct validity and be sensitive to change using the data from a recently conducted clinical trial [15], and has demonstrated reliability in a separate study [14], reflective of the construct validity and responsiveness of the full NPCCSS measure [7, 11, 12]. The high correlation reported by Cortina-Borja et al. 2018 [8] between the 5-domain NPCCSS and the 17-domain NPCCSS was also confirmed (correlation = 0.97), providing data to support the criterion validity of the 5-domain NPCCSS.

Interpretation of any outcome measure score is essential to allow understanding of whether observed changes in a condition are meaningful or not. There has been no prior research looking at this specific element of the NPCCSS measure. Data gathered in both the qualitative studies and the clinical trial provided evidence that a 1-category change in any domain, corresponding to a 1–2-point change in total score of the 5-domain NPCCSS scale, is meaningful. This indicates that worsening by 1 category is a reflection of loss of complex function and increased disability.

Among caregivers and patients across all levels of severity in each domain, all but one participant reported a 1- or 2-category worsening as being meaningful, with most stating a 1-category worsening is meaningful. Clinicians agreed that the measure captures clinically important and relevant NPC symptoms and domain changes, and that delaying progression was important.

The qualitative data provide support and contextualization to the quantitative approaches to determine the minimal clinically important difference for the 5-domain NPCCSS. The anchor-based analyses suggested that progressing beyond a 1-point worsening on the 5-domain NPCCSS would be clinically meaningful and, therefore, preventing a 2-point worsening would be a viable treatment goal.

A potential limitation to the mixed methods study is the small sample size for the web-based survey (n = 49) and interviews (n = 28), especially when considering the broad age range of patients (1–65 years). While there was a good proportion of adult and paediatric patients represented, there was a limited number of adults reporting for themselves (web-based survey n = 6; interviews n = 5) (which could reflect the nature of the condition), and also a small number of very young patients included (aged 6 months to 3 years; web-based survey n = 5 and interviews n = 1). Although the key domains appeared to be generally consistent across age groups in the current survey, there may be some differences in the very young children where Fine Motor Skills were not always ranked as most important. In addition, swallowing impairment tends to begin later and occur when the disease has progressed in severity. While the 5 domains are relevant for all age groups, it is acknowledged that the assessments have some limitations for the very young (0–2 years) age group. For example, ambulation impairments may be difficult to assess in the very young who would not yet be expected to have reached this developmental milestone. An alternative age appropriate measure would need to be utilized for this age group. Furthermore, the interviews focused on those domains highlighted as important or within the 5-domain NPCCSS which means that some of the original 9 domains were not discussed in depth, however the findings suggest that the domains focused on were those that were most important. A further potential limitation is that the patient or caregiver interviews and survey were only conducted in 2 countries (US and UK). Research involving participants from other countries would enable confirmation of cross-cultural validity. Finally, although the qualitative and quantitative data support each other regarding what constitutes a meaningful worsening on the 5-domain NPCSS (ie, 1-point or greater) the gold-standard, anchor-based approach, was based on a small group of subjects (n = 13) due to the late introduction of CGI into the study. Furthermore because of the late introduction, there was increased risk of recall bias as the ratings for the majority of patients were based upon retrospective evaluations of disease status at baseline. Therefore, it is recommended that future researchers continue to explore this property of the measure.

## Conclusions

The results of the mixed methods, qualitative studies and clinical trial data are supportive of the 5-domain NPCCSS as a valid measure of NPC. Importantly, through this research, a meaningful change threshold has also been proposed. Nearly all participants expressed in interviews that slowing the progression within any domain on the 5-domain NPCCSS scale would be meaningful to patients and/or caregivers, and this was echoed by clinicians. A 1-category change on a domain equivalent to 1-point or greater change in the 5 domain NPCCSS total score supports a clinically meaningful transition. This supports that each category reflects loss of complex function and increased disability. The anchor-based analyses suggest that progressing beyond a 1-point worsening on the 5-domain NPCCSS would be clinically meaningful and, therefore, preventing a 2-point worsening would be a viable treatment goal.

The reliability has also been demonstrated both within clinician (intra-rater) and between clinicians (inter-rater) [14]. This evidence is supportive of the 5-domain NPCCSS as a useful measure for assessing disease progression in NPC patients.

## Methods

### NPC patient survey (OR-SRV-NPC-01)

This study involved adult patients (aged ≥ 18 years) and caregivers of either paediatric (< 18 years old) or adult patients from the US and UK with a confirmed diagnosis of NPC. There were 2 parts to the study: Part 1 was a web-based survey; and Part 2 was a follow-up telephone interview with a subset of survey participants.

In Part 1, participants were asked to rate the severity of their NPC (or the NPC of the person under their care) using a series of questions based upon the 9 major domains of the NPCCSS (Ambulation, Speech, Swallow, Fine Motor Skills, Cognition, Memory, Seizures, Hearing, Eye Movements). The NPCCSS questions and response options had been reworded to be appropriate completion by patients and caregivers while still reflecting the clinician-reported version of the NPCCSS. Definitions for some of the domains were provided. For example, for Cognition, participants were directed to think of cognitive ability as the ability to learn new skills, make decisions, follow instructions, or focus attention; within the measure this is considered distinct from Memory. The 5-domain NPCCSS scores were then used to select a range of severities for the interviews. Participants were also asked to identify and rank the 5 most important domains in NPC from 1 = “the very most important symptom” to 5 = “the least important symptom.” They were asked to identify these from the 9 major domains or given the option to add others.

In Part 2, semi-structured interviews (approximately 90 min long) were conducted by trained interviewers following a discussion guide. All were conducted in English. Although the survey asked about the 9 major domains of the NPCCSS, the interviews focused on exploring the 5-domain NPCCSS symptoms and any other symptoms that participants had indicated as being most important in the web survey. Participants were asked why these symptoms were important, how they impacted activities of daily living (ADL) and health related quality of life (HRQoL), what category decline in each domain would be meaningful (from the point of their current level, a 1-category decline would be a movement to the next more severe response description on an item), and what such a change would mean in terms of impact on ADLs and HRQoL. The response option range of the 5 domains are as follows: Ambulation (normal—wheelchair dependent); Speech (normal—minimal communication); Swallow (normal – nasogastric tube feeding only); Fine Motor Skills (normal—severe dysmetria/dystonia [gross motor limitations, requires assistance for self-care activities]); and Cognition (normal—minimal cognitive function). This information was used to infer what change in total score (range 0–25) on the 5-domain NPCCSS would be meaningful, since it is cognitively challenging to qualitatively discuss the meaning of change at the overall scale level with patients/caregivers.

### NPC expert clinician interviews

In-depth, semi-structured interviews lasting up to 1 h were conducted with clinician experts over the telephone. The interviews explored the importance and relevance of the 5 domains, the level of change clinicians consider to be meaningful in the 5-domain NPCCSS score, and each of the separate domains.

### NPC clinical trial (CT-ORZY-NPC-002)

The construct validity and meaningful change threshold for the NPCCSS was derived from a 12-month, prospective, randomized, double-blind, placebo controlled therapeutic study in paediatric patients with a confirmed diagnosis of NPC (NCT02612129) [15]. A total of 50 subjects were randomized and received either placebo (n = 16) or arimoclomol (n = 34). The age range was 2–19 years (mean [SD] 11.1 years [5.0]) and there were 26 females and 24 males.

Convergent validity (construct validity) was assessed using Spearman’s correlation coefficient due to the categorical nature of the response options. A treatment effect was evaluated using mean change (SD) from baseline across both treatment arms (placebo and arimoclomol). Analysis of meaningful change through the anchor-based approach used the CGI-I as the anchor [18]. The CGI-I is a 1-item measure of change: “Rate total improvement whether or not, in your judgement, it is due entirely to drug treatment” with a 7 category response scale ranging from “very much improved” to “very much worse.”

The CGI-I was collected throughout the trial by the same investigator for each participant. To determine the anchor-based meaningful change threshold, patients were grouped by CGI-I levels. Mean and median change, 95% confidence interval, and standard deviation of the NPCCSS were calculated by CGI-I category.

The analysis included all patients, independent of the assigned treatment group, and was performed on blinded data.

## Data Availability

The datasets generated and/or analyzed during the current study are not publicly available, but are available from the corresponding author on reasonable request.

## Abbreviations

ADL: Activities of daily living
CI: Confidence interval
CGI-I: Clinical Global Impression Scale-Improvement
CGI-S: Clinical Global Impression Scale-Severity
HRQoL: Health related quality of life
MCT: Meaningful change threshold
NPC: Niemann-Pick disease type C
NPCCSS: Niemann-Pick type C Clinical Severity Scale
SARA: Scale for Assessment and Rating of Ataxia
SD: Standard deviation
